# A Protocol for Microprism‐Based Two‐Photon Imaging Of The Lateral Cortex In The Mouse Inferior Colliculus

**DOI:** 10.1002/cpz1.70286

**Published:** 2025-12-31

**Authors:** Baher A. Ibrahim, Daniel A. Llano

**Affiliations:** ^1^ Department of Molecular & Integrative Physiology University of Illinois at Urbana‐Champaign Urbana‐Champaign Illinois; ^2^ Beckman Institute for Advanced Science & Technology University of Illinois at Urbana‐Champaign Urbana‐Champaign Illinois; ^3^ Neuroscience Program University of Illinois at Urbana‐Champaign Urbana‐Champaign Illinois; ^4^ Carle Illinois College of Medicine University of Illinois at Urbana‐Champaign Illinois United States

**Keywords:** GABAergic modules, Inferior colliculus, lateral cortex, microprism, two‐photon imaging

## Abstract

The inferior colliculus (IC) is a central hub for auditory information processing that receives widespread convergent projections. The IC comprises three main subdivisions: the central nucleus of the IC (ICC), the dorsal cortex (DC), and the lateral cortex (LC). While the ICC receives primarily ascending auditory information, DC and LC receive major cortical and multisensory projections. The LC has repeated molecular motifs that govern its input‐output relationships. However, because the LC is buried deep within a sulcus, it is difficult to image in behaving animals, making it challenging to answer questions about its functional organization. Here, we describe a protocol for coupling two‐photon microscopy with a microprism to obtain cellular‐resolution sagittal views of functional LC maps. We employed this novel approach to investigate neuronal responses to pure tones in relation to LC motifs. This method will not only provide new insights into the auditory system but will also permit imaging of hidden brain regions previously inaccessible by conventional means. © 2025 The Author(s). *Current Protocols* published by Wiley Periodicals LLC.

**Basic Protocol 1**: Craniotomy and implantation of the microprism.

**Basic Protocol 2**: Data acquisition from sound‐responsive neurons.

**Basic protocol 3**: Confirming the microprism location.

**Basic Protocol 4**: Analyzing the time traces of neuronal responses and generating a best‐frequency tuning Map.

## Introduction

The inferior colliculus (IC) is a critical integration center in the auditory system (Ito & Oliver, 2012). The IC comprises three major divisions including the central nucleus (ICC), which receives primarily ascending auditory projections (Oliver, 2005; Willott, 2001; Willard & Ryugo, 1983) as well as the dorsal (DC) and lateral (LC) cortices that receive massive descending auditory as well as multisensory input (Bajo & Moore, 2005; Coleman & Clerici, 1987; Loftus et al., 2008; Saldaña et al., 1996; Schreiner & Winer, 2005; Winer et al., 1998). The LC and DC appear to differ in their strategy for integrating these inputs (Ibrahim et al., 2025). The DC lacks known structural heterogeneities but exhibits a tonotopic organization of neuronal responses on its surface (Barnstedt et al., 2015; Ibrahim et al., 2025; Ito et al., 2014; Wong & Borst, 2019). In contrast, the LC contains repeated neurochemical motifs, here referred to as modules, that stain intensely for the inhibitory neurotransmitter GABA and other metabolic markers (Chernock et al., 2004). Areas outside the modules, referred to as matrix, stain strongly for calretinin (Dillingham et al., 2017). We and others have determined that nearly all of the LC's long‐ and short‐range connectivity is governed by the module/matrix organization (Dillingham et al., 2017; Lamb‐Echegaray et al., 2019; Lesicko et al., 2016, 2020). Unfortunately, how LC integrates these inputs while maintaining distinct paths of information flow is not known. For example, LC neurons are known to respond to multisensory stimuli (Jain & Shore, 2006; Zhou & Shore, 2006), but information from different modalities enters the LC in segregated channels (Lesicko et al., 2016). Some crosstalk between the matrix and modules has been observed (Lesicko et al., 2020), but the mechanisms underlying this integration are not fully understood. Traditional methods of studying the LC, such as multichannel electrophysiology, pose significant challenges for unambiguously assigning individual recorded neurons to the matrix or modules. Imaging approaches, such as two‐photon (2P) microscopy, have proven to be invaluable for understanding the fine spatial structure of neural response properties in the auditory system (Bandyopadhyay et al., 2010; Romero et al., 2020; Rothschild et al., 2010). However, because of its superficial location on the dorsal surface of the mouse midbrain, the DC is the only part of the IC that has been characterized by 2P imaging (Barnstedt et al., 2015; Ito et al., 2014; Wong & Borst, 2019). Optically, it has been impossible to characterize the functions of the LC, which is laterally embedded deep in a brain sulcus. This inability to image the LC impedes our understanding of how the LC performs the critical tasks of segregation and integration, whether neurons within modules or in the surrounding matrix exhibit different response properties, and whether tonotopy or other spatial structures in response properties exist. Our recent work showed that 2P images obtained with the microprism yielded the first in vivo images of the LC, revealing its characteristic GABAergic modules. Also, acoustically evoked calcium signals recorded by 2P imaging of the LC through the microprism revealed functional distinctions between modules and the matrix (Ibrahim et al., 2025). Here, a detailed protocol is provided to describe the novel approach, which uses a 45 ° microprism inserted into the sulcus separating the LC from the cerebellum. This method investigates the fine‐scale functional organization of the LC from its lateral surface through 2P imaging. The protocol also describes image acquisition and analysis, as well as the validation of the technique. This novel method will offer neuroscientists a new experimental approach to optically examine and manipulate the auditory and non‐auditory functions of the LC, its inputs, or other brain regions hidden in sulci or other inconvenient locations.

*NOTE*: All work described in this protocol was approved by the University of Illinois at Urbana‐Champaign Institutional Animal Care and Use Committee. All protocols involving animals must be reviewed and approved by the appropriate local Animal Care and Use Committee and must follow regulations for the care and use of laboratory animals.

## Basic Protocol 1 CRANIOTOMY AND IMPLANTATION OF THE MICROPRISM

In this protocol, we demonstrate the surgical procedures for the craniotomy over the surface of the IC as a first step before microprism implantation. The second step outlines the procedure for inserting the 45 ^°^ microprism into the sulcus between the IC and the cerebellum, ensuring that the microprism's orthogonal side faces the lateral surface of the IC.

### Materials

Surgical tools
  Feather scalpel blade #10
  Number 5/45 forceps (F.S.T., Germany, cat. no. 11251‐35**)**
  Dumont #5SF Forceps (F.S.T., Germany, cat. no. 11252‐00)
  Dumont #5 Forceps (F.S.T., Germany, cat. no. 11251‐20)
  A micro drill bit (size #80, Grainger, USA)
  Bonn microprobe (F.S.T., Germany, cat. no. 10032‐13)
  Fine Scissors – Sharp (F.S.T., Germany, cat. no. 14060‐11)
  30° diamond knife (F.S.T., Germany, cat. no. 10100‐30)
Glass items
  45° microprism (1.5 mm, silver‐coated, Tower Optical Corporation)
  5 mm cover glass #1 (Thomas Scientific, USA)
Animals
  GAD67‐GFP/CBA/RGECO1a mouse (Produced by 10 generations of back‐crossing [(GAD1GFP) knock‐in mouse × CBA/CaJ (Graves et al., 2025)] and Tg(Thy1‐jRGECO1a)GP8.20Dkim/J mouse (Jackson Laboratory, Stock# 030525)
  The GAD1GFP knock‐in mouse was developed and shared with permission from Dr. Yuchio Yanagawa at Gunma University and obtained from Dr. Douglas Oliver at the University of Connecticut, where GFP is exclusively expressed in GABAergic cells (Tamamaki et al., 2003).
Devices
  Autoclave
  59S UV‐C UV LED sterilizer sanitization box (Walmart, USA)
  Scale for weighing the animal
Medications
  0.9% Sodium Chloride (Fisher Scientific, NC9054335)
  Dexamethasone Injection (2 mg/ml) (PetSmart, 5317174) (Dose: 5 mg/kg, Concentration: 0.5 mg/ml)
  Ketamine/Xylazine/Acepromazine mixture (10 ml)
  a) 1 ml of 100 mg/ml Ketamine Hydrochloride Injection (Covetrus, 080524) to make stock solution of 10 mg/ml, given at a dose of 100 mg/kg.
  b) 0.15 ml of 20 mg/ml of Rompun (Xylazine Injection) (Covetrus, 080907) to make stock solution of 0.3 mg/ml, given at a dose of 3.0 mg/kg.
  c) 0.15 ml of 10 mg/ml Acepromazine Injectable Solution (Covetrus, 003845) to make stock solution of 0.15 mg/ml, given at a dose of 1.5 mg/kg.
  d) 8.7 ml saline for injection
Ketamine solution
  a) 1 ml of 100 mg/ml Ketamine Hydrochloride Injection (Covetrus, 080524) to make stock solution of 10 mg/ml, given at a dose of 100 mg/kg.
  b) 9.0 ml saline for injection
  Carprofen Injectable Solution (Covetrus, 059149) (Dose: 5 mg/kg, concentration: 0.5 mg/ml in sterile saline).
  Lidocaine 2% Injection in sterile saline (Allivet, USA)
  Povidone Iodine Swabsticks (Dynarex, USA)
Miscellaneous
  Metabond cement kit (Parkel, USA)
  Sterile cotton‐tipped applicators (Medline, USA)
  Hemostatic sponge (Hemosponge, Goodwill, India)
  Ice bucket
  Shaving cream (Nair)
  Eye lubricant (Optixcare)
  Agarose (Sigma‐Aldrich)
  Customized headpost
  Timer
  Heating pad with a feedback thermometer (FHC, USA)

#### Preparation for surgery

1 All surgical procedures involving mice must be reviewed by the relevant local institutional animal care and use committee.
2 Sterilize all surgical tools in the autoclave for at least 30 min at 121°C the day before surgery to allow complete drying.
3 On the day of surgery, place the tools next to the sterilized platform, adjacent to the stereotaxic apparatus.
4 The diamond knife cannot be autoclaved due to cracking of the diamond piece. Instead, the diamond knife and microprism must be sterilized using UV‐C light (59S UV‐C UV LED sterilizer sanitization box).
5 Prepare all drugs to the specified concentration.
6 In the ice bucket, keep a ceramic holder (a component of the *C&B Metabond kit)* for later preparation of the dental cement mixture.
7 In sterile saline solution, cut a sterile hemostatic sponge into small pieces (∼5–10 mm) and keep it in its holder on ice.
8 Keep the carprofen and lidocaine on ice during the surgery.
9 Keep a stream of cold air and an ice‐cold saline solution ready for use.

#### Craniotomy over the IC surface

**Figure 1:**
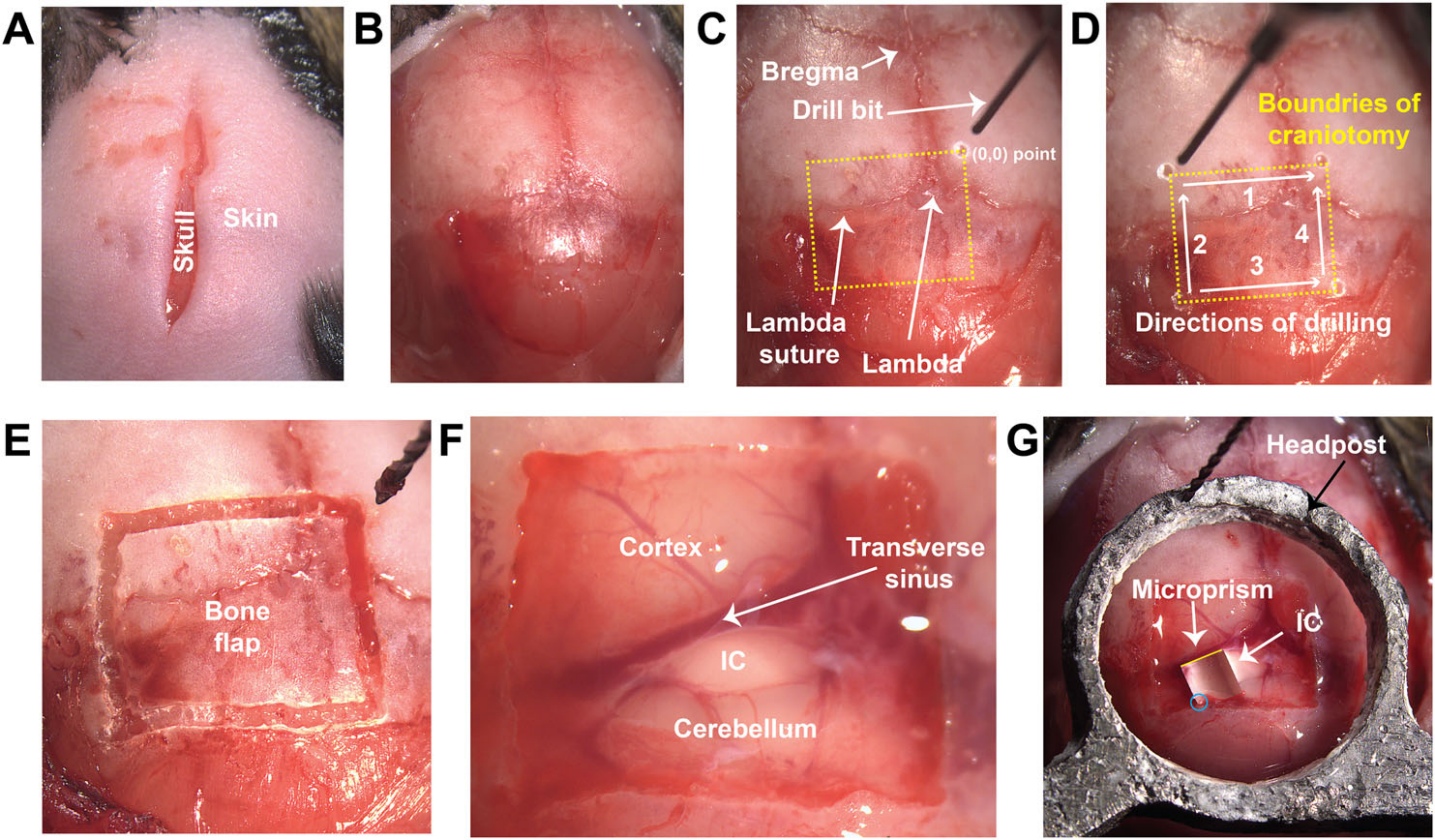
Craniotomy surgery over the IC surface. Micrographs demonstrate (**A**) a longitudinal incision made in the skin over the mouse's skull; (**B**) the mouse's skull after removing the whole skin and periosteum membrane; (**C**) the drilling start point; (**D**) the boundaries of the cranial window and the directions of drilling. (**E and F**) Micrograph showing the mouse's skull before and after removing the bone flap, as well as the brain structures underneath, respectively. (**G**) A micrograph shows the location of the microprism on the IC's lateral side. The blue circle indicates where the microprism can be secured under the skull, providing more stability.

10 Gently remove the mouse from its cage and weigh it. Record the animal's weight in the surgical report in grams. Based on this weight, calculate the volume of drugs needed.
11 Inject the animal with an appropriate volume of Ketamine/Xylazine/Acepromazine (0.1 ml per 10 g of the animal's weight) intraperitoneally.
12 After injection, return the animal to its cage and observe until there is no noticeable response to a toe pinch. If the animal needs more anesthesia, inject with an extra dose of ketamine‐only solution (25% of the initial dose in Ketamine/Xylazine/Acepromazine mixture), with frequent checking for the lack of toe response to pinching.
13 Secure the animal to the stereotaxic apparatus and make sure the skull is in its horizontal plane between the nose and ear bars. Immediately apply an eye lubricant.
14 Put the animal above the heating pad to maintain body temperature at 32°C to 36°C.
15 To remove hair from the skull, apply hair removal cream and gently massage the skin using a small cotton‐tipped applicator. After 5 min, scrape away the cream with the removed hair. Clean the shaved skin with povidone alternating with 70% ethanol.
16 Using scalpel No. 10, make a longitudinal incision (Fig. 1A).

17 Apply 0.3 ml of 2% lidocaine intradermally to the incised area and wait for 1 min.
18 With forceps and scissors, cut the skin completely off the shaved area to permit complete access to the skull. Then, with the scalpel, remove all the periosteum completely from the surface of the skull (Fig. 1B).
19 Dry the skull with a cotton‐tipped applicator and then apply universal dentin activator gel (a component of the Metabond kit) for 10–30 s to prepare the skull for headpost implantation. Wash the gel off with saline and dry the skull with a cotton‐tipped applicator.
20 From lambda, move the drill bit 1.0 mm rostrally and 0.8 mm laterally to the right hemisphere, where the first drilling takes place, and assign this point as the start location (x = 0, y = 0, Fig. 1C).
21 From the starting point, locate the borders of the cranial window by drilling at three additional spots, as follows: (x, y) = (0, 3.5), (4, 3.5), and (4, 0) mm (Fig. 1D).
22 Drill through the skull to create grooves connecting the four drilled spots following the directions of drilling, resulting in a final rectangular shape that centralizes the IC surface (Fig. 1E). To minimize bleeding, apply cool air and cold saline during the drilling process.
  This drilling approach minimizes the risk of bleeding.
23 Once you have the rectangular shape, drill any area of the bone flap that is still attached to the surrounding skull. Complete detachment is confirmed by gently pressing down on the bone flap (Video 1).

**Video 1:** A short movie shows a mechanism of gently pressing down on the bone flap to confirm its complete detachment from the skull.

24 Once you have a loose bone flap, apply extra cold saline to the area and lift the detached bone flap with Dumont #5 forceps. You may expect some bleeding due to the detachment between the bone flap and the underlying dura. If this happens, immediately apply a saline‐soaked hemostatic sponge to the bleeding areas and dry with a cotton‐tipped applicator. You may repeat this process until the bleeding stops. Keep the area as wet as possible with saline. After removing the bone flap and controlling any potential bleeding, the brain structures underneath should appear clean, with no blood clots or bleeding (Fig. 1F).
25 After obtaining a clean brain surface with no blood, gently poke the dura above the midline without puncturing the underlying venous structures with a sterile, sharp 27G needle tip. Then, insert the tip of the Bonn microprobe and gently lift the dura to tear it over the surface of the IC (Video 2). You may use the Dumont #5SF forceps to remove the remnants of dura at the borders of the IC surface, especially at the lateral side where the microprism is implanted.
  Do not attempt to peel the dura from above the transverse sinus. Peeling the dura at this location could result in a severe cut of the sinus and serious bleeding.

**Video 2:** A short movie shows two steps of removing the dura from above the IC surface.

#### Microprism implantation at the lateral surface of the IC

26 Install the diamond knife holder and set its tip at the midline of the IC. Then move the knife laterally for 1.5 mm, which is the minimum distance after which the knife should be inserted (Video 3).

**Video 3:** A short movie shows a complete procedure of microprism implantation at the lateral cortex of the IC.

27 Select an area free of blood vessels and drive the knife ventrally until it touches the surface of the IC. Then, zero out the z‐axis of the stereotaxic apparatus. Place the prism at the front of the knife, ready for installation.
28 Insert the knife 1.5–1.8 mm ventrally. Then, move the knife medially by 0.3–0.5 mm to create a pocket (Video 3).
  The knife has two surfaces (engraved and flat). Both surfaces must be pristine, with no residues or scratches. The flat surface should face the IC tissue, and the engraved surface should face the microprism (Video 3).
29 Maneuver the prism so its orthogonal side faces the knife's surface, allowing you to see it through the prism's top surface (Video 3).
30 Using a pair of forceps, secure the prism in place by pressing down on the top surface of the microprism. Retract the knife upward, continuing to press the microprism down. This synchronized motion will prevent the microprism from rising due to tissue pressure beneath it. It will also cause the lateral tissue, initially pushed by the knife, to return to its original position, facing the prism's orthogonal surface (Video 3).
31 Hold the prism in position by keeping the knife on the surface for at least 10 min. During this period, control any bleeding around the microprism with saline. In the meantime, prepare a 1.2% agarose gel.
  If the microprism is close to the bottom edge of the rectangular cranial window, you may try to secure the bottom right corner of the microprism underneath the skull, providing more stability for the microprism in its place (Fig. 1G, blue circle).
32 Prepare the agarose gel by mixing 10 ml sterile saline and 0.12 g agarose. Then microwave the mixture for 25 s and cool the clear gel to 37°C.
33 Remove the diamond knife from the area and place a 5‐mm coverslip over the whole cranial cavity, including the prism and IC surface.
34 Using a Pasteur pipette, inject the agarose gel in its liquid form beneath the coverslip, allowing it to take the shape of the cavity beneath the glass and secure the microprism in place.
35 Remove the extra agarose from around the window or above the surrounding skull.
36 Secure the head post so that its circular shape surrounds the cranial window, and make sure the horizontal surface of the head post is on the same level as the cover glass of the cranial window (Fig. 1G).
37 In an ice‐cold ceramic chamber, mix the components of the Metabond dental cement according to the kit instructions. In brief, mix 4 drops of a quick base for C&B Metabond with 1 drop of universal TBB catalyst and 2 scoops of radiopaque L‐Powder for C&B Metabond until a homogeneous paste forms.
38 Apply the liquid paste to the boundaries of the cover glass to secure it to the skull and the metal head post. Apply the paste to secure the headpost to the skull.
39 Wait until the cement is strong enough to secure all the components over the skull.
40 Inject the animal with 5 mg/kg carprofen and keep it on a heating pad until the imaging session.

## Basic Protocol 2 DATA ACQUISITION FROM SOUND‐RESPONSIVE NEURONS

In this protocol, we demonstrate how to set up the animal under the microscope to image the LC using a microprism and to record calcium signals from live neurons following pure‐tone presentation.

### Materials

A mouse ready for imaging
Two‐photon system:
  Laser system: Insight X3, Spectraphysics
  Olympus microscope
  Brucker optical components
  Prairie View software (version 5.8)
  Photomultiplier tube (Hamamatsu H7422PA‐4, Japan)
  t565lp dichroic
  Chroma barrier et525/70m filter for GFP
  et595/50m filter for jRGECO1a signals.
Objectives
  4× air fluorescent objective (Olympus, UplanFI)
  16× water‐immersion objective (N16XLWD‐PF ‐ CFI LWD Plan Fluorite Objective, 16×, NA: 0.8, WD: 3.0 mm; Nikon, Tokyo, Japan)
Miscellaneous
  CoolSNAP MYO camera
  Lumen 200 bulb (Prior Scientific Inc, USA)
  Heating device with a feedback thermometer
  Wavelength Multi‐Purpose Ultrasound Gel (National Therapy Product Inc., Canada).

#### Setting up the animal under a 2P‐microscope system

**Figure 2:**
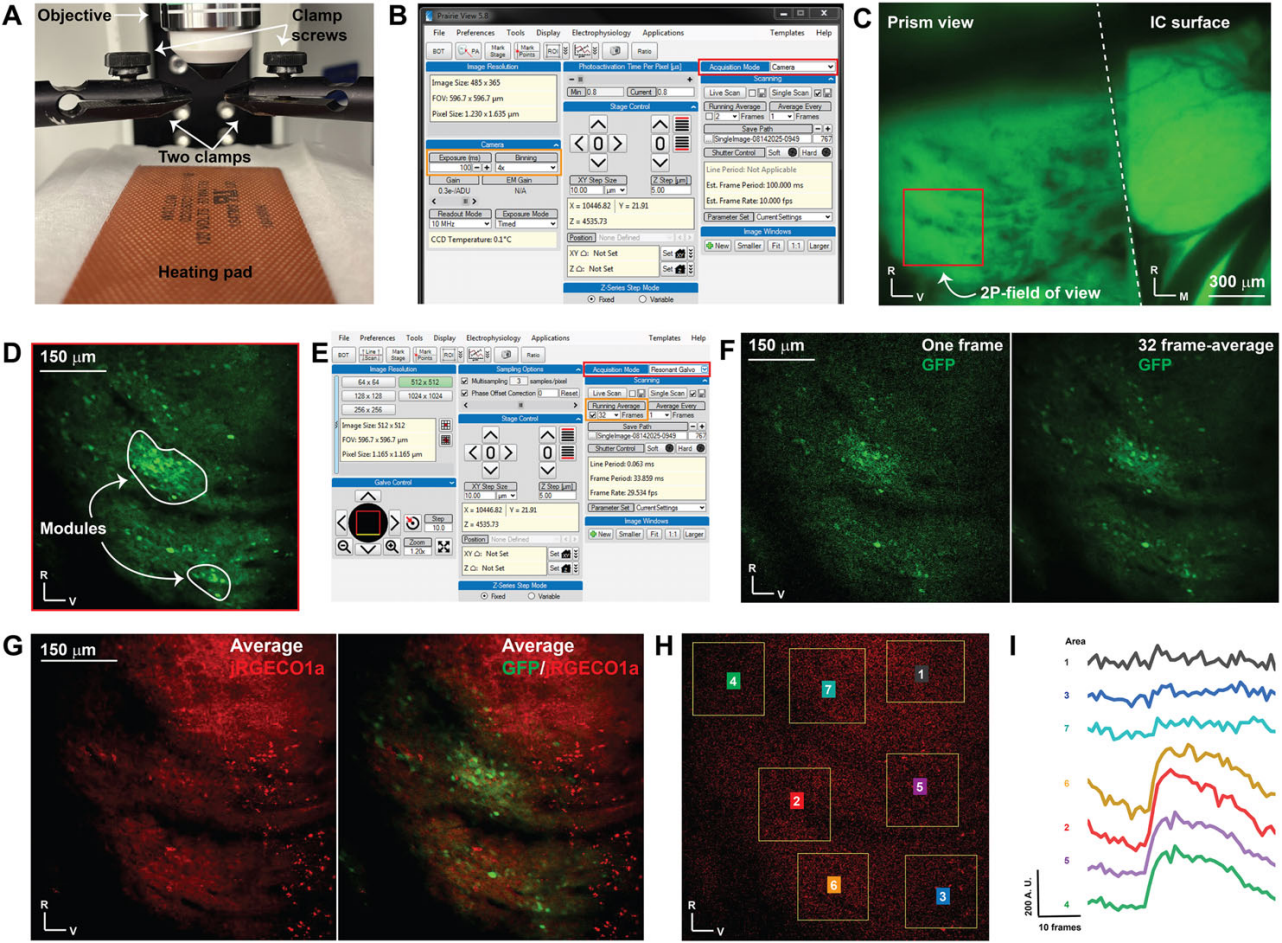
Setting up the mouse for imaging through the microprism. (**A**) A micrograph showing the setup under the microscope objective. (**B**) A screenshot of the Prairie View interface window showing the setup to capture an epifluorescent image in the camera acquisition mode (red box), as well as the binning and exposure time controllers (orange box). (**C**) An epifluorescent image showing the IC top surface and the prism view of the LC, indicated by the GFP signals. (**D**) A 2P image under a galvo acquisition mode of the LC showing the GABAergic modules. (**E**) A screenshot of the Prairie View interface window showing the setup in resonant galvo acquisition mode (red box) to capture 2P images and how to deploy a running average of the live view (orange box). (**F**) 2P images in resonant galvo mode showing the GABAergic modules by a single frame vs a running average image of 32 frames. (**G**) 2P images in resonant galvo mode showing the Jrgeco1a expressing cells along with GABAergic modules. (**H**) The whole field of view is divided into different areas to be examined for sound response. (**I**) The time traces of the population response to the search stimulus (broadband noise at 80 dB) across different areas of the field of view are shown in H.

1 Place the mouse on a heating pad and maintain its body temperature between 32°C and 37°C.
2 Secure the two arms of the head post to the corresponding clamps, so the head of the animal will be under the objective position (Fig. 2A).

3 Tighten the screws of the clamp to secure the head of the animal firmly under the objective lens, thereby reducing the motion artifact (Fig. 2A).
4 With Prairie View software, use the camera acquisition mode (Fig. 2B, red box) to examine the quality of the window and verify there are no signs of bleeding or light‐blocking blood clots by imaging the GFP fluorescence signals in GAD67‐GFP/CBA/jRGECO1a mice using low magnification (4× Olympus objective, 4 × 4 binning, 100 ms exposure time) (Fig. 2B, orange box) with a Lumen 200 bulb and CoolSNAP MYO camera (Photometrics, USA, excitation: 488 nm and emission: 515–550 nm). The epifluorescence image of GFP signals shows the top surface of the IC on the right, and the image obtained with the microprism on the left (Fig. 2C).
5 Prepare the aqueous gel for the immersion objective by mixing one part Wavelength Multi‐Purpose Ultrasound Gel with two parts double‐deionized water. Apply the gel above the window.
6 Using a water‐immersed 16× objective with a 3 mm working distance, attempt to obtain a focused view of the prism surface using the GFP fluorescence signals as a marker, following the exact excitation and emission parameters. The goal is to obtain a view of the center of the microprism's top surface.

#### Recording calcium signals following a sound playback

7 To collect a high‐resolution image using the 2P configuration, switch to galvo acquisition mode in Prairie View. Then turn on the laser and open its hardware shutter.
8 Select the 920 nm excitation laser and a suitable PMT to detect GFP emission using a Chroma barrier et525/70m filter.
9 Adjust the laser power and PMT gain to avoid over‐ and undersaturation, ensuring an image of the LC surface with its characteristic GABAergic modules as indicated by the GFP signals (Fig. 2D).
10 To collect calcium signal data, switch to the resonance galvo acquisition mode and adjust the laser power and PMT gain accordingly (Fig. 2E, red box).
  Since the resonant galvo mode is a high‐speed acquisition mode, cell searches should be performed with a running average of at least 32 frames (Fig. 2E, orange box). Figure 2F shows the difference between a single frame and the view of a 32‐frame average of the same cells.
11 Once you have attained a focused field of view of the GABAergic cells via GFP signals, switch to a 1040 nm laser along with a 595/50m filter to obtain an image of the jRGECO1a‐expressing cells (Fig. 2G).
  Because the jRGECO1a is driven via the Thy1 promoter, the calcium indicator is only expressed in the non‐GABAergic cells (Fig. 2G‐left).
12 Test the selected field of view for sound responsivity by presenting the search‐sound stimulus (broadband noise at 80 dB SPL). First, use the BOT function to draw multiple rectangular ROIs that cover the entire field of view (Fig. 2H). Using the time series mode, capture a video of the jRGECO1a signals during the presentation of the test sound. In the BOT real‐time signals, a peak in the calcium signal observed at any of the drawn ROIs indicates the presence of a sound‐responsive area (Fig. 2I).
13 Within the sound‐responsive areas, take a series of videos of different combinations of pure tone frequencies and amplitudes in a pseudorandomized order. In this protocol, we used seven pure tone frequencies (5, 7.1, 10, 14.1, 20, 28.3, and 40 kHz) and five amplitudes (40, 50, 60, 70, and 80 dB SPL). Therefore, each movie has 35 combinations in a different pseudorandomized order. Each combination lasts 500 ms, with an ISI of 1.1 s (onset‐to‐onset).
  In our setup, the TDT ES1 speaker output was calibrated using a PCB 377A06 microphone, which feeds a SigCal tool to generate a calibration file for all tested frequencies (5–40 kHz). To enable the custom‐made MATLAB code to read this calibration file, the values were first processed by MATLAB signal processing toolbox (sptool) to generate a 256‐tap FIR filter to apply the calibration using the following parameters [arbitrary magnitudes, least square, order: 256, sampling rate: 97,656.25, frequency vector (5‐40 kHz), amplitude vector (40‐80 dB SPL), and weight vector ones (1128)].
14 The 35 frequency/amplitude combinations should be distributed randomly in the sound file. Using the time‐series mode, capture a 40‐s movie for each sound file and save each as an image sequence in its corresponding directory. Each movie is assigned a name according to the key of the order of the frequency/amplitude combinations used as stimuli to generate that movie.

## Basic Protocol 3 CONFIRMING THE MICROPRISM LOCATION ADJACENT TO THE LC

In this protocol, we demonstrate how to confirm the microprism's location on the LC surface. By the end of data acquisition, we lesion the field of view with a high‐power laser, allowing the area imaged via the microscope to be examined histologically to locate the laser lesion. This step is considered the final procedure after fulfilling all required imaging requirements and addresses the research question.

### Materials

The mouse used for 2P imaging and data acquisition.
Two‐photon system:
  Laser system: Tunable laser (700–1300 nm) ‐ Insight X3, Spectraphysics
  Olympus microscope
  Brucker optical components and Prairie View software
  Photomultiplier tube (Hamamatsu H7422PA‐4, Japan)
  t565lp dichroic
  Chroma barrier et525/70m filter for GFP
  et595/50m filter for jRGECO1a signals
Objectives
  4× air fluorescent objective (Olympus, UplanFI)
  16× water‐immersion objective (N16XLWD‐PF ‐ CFI LWD Plan Fluorite Objective, 16×, NA: 0.8, WD: 3.0 mm; Nikon, Tokyo, Japan)
Surgical tools
  Dumont #5 Forceps (F.S.T., Germany, cat. no. 11251‐20)
  Fine Scissors – Sharp (F.S.T., Germany, cat. no. 14060‐11)
  Student Hose Clamp (F.S.T., Germany, cat. no. 91198‐15)
  Dissector Scissors (F.S.T., Germany, cat. no. 14082‐09)
*Since these surgical tools may come into contact with PFA, they are part of a separate kit and are not used for surgery*.
Chemicals
  1× PBS (Fisher Scientific, USA)
  4% PFA (Fisher Scientific, USA)
  One egg, raw
  Sucrose (Sigma Aldrich, USA)
  O.C.T. Compound (Scigen Scientific Gardena, USA)
  Vectashield mounting medium with DAPI (Cole Palmer, USA)
Miscellaneous
  CoolSNAP MYO camera
  Heating device with a feedback thermometer
  Wavelength Multi‐Purpose Ultrasound Gel (National Therapy Product Inc., Canada).
  27G needle
  Small glass container to keep the brain for PFA fixation
  Glassware for chemical preparation
  Coverslips (Warner Instruments, USA)
  Microscopical slides (Fisher Scientific, USA)
  Cryostat (Liyca, USA)
  Single‐photon fluorescence upright Olympus microscope

#### Laser lesioning of the lateral surface of the IC using microprism

**Figure 3:**
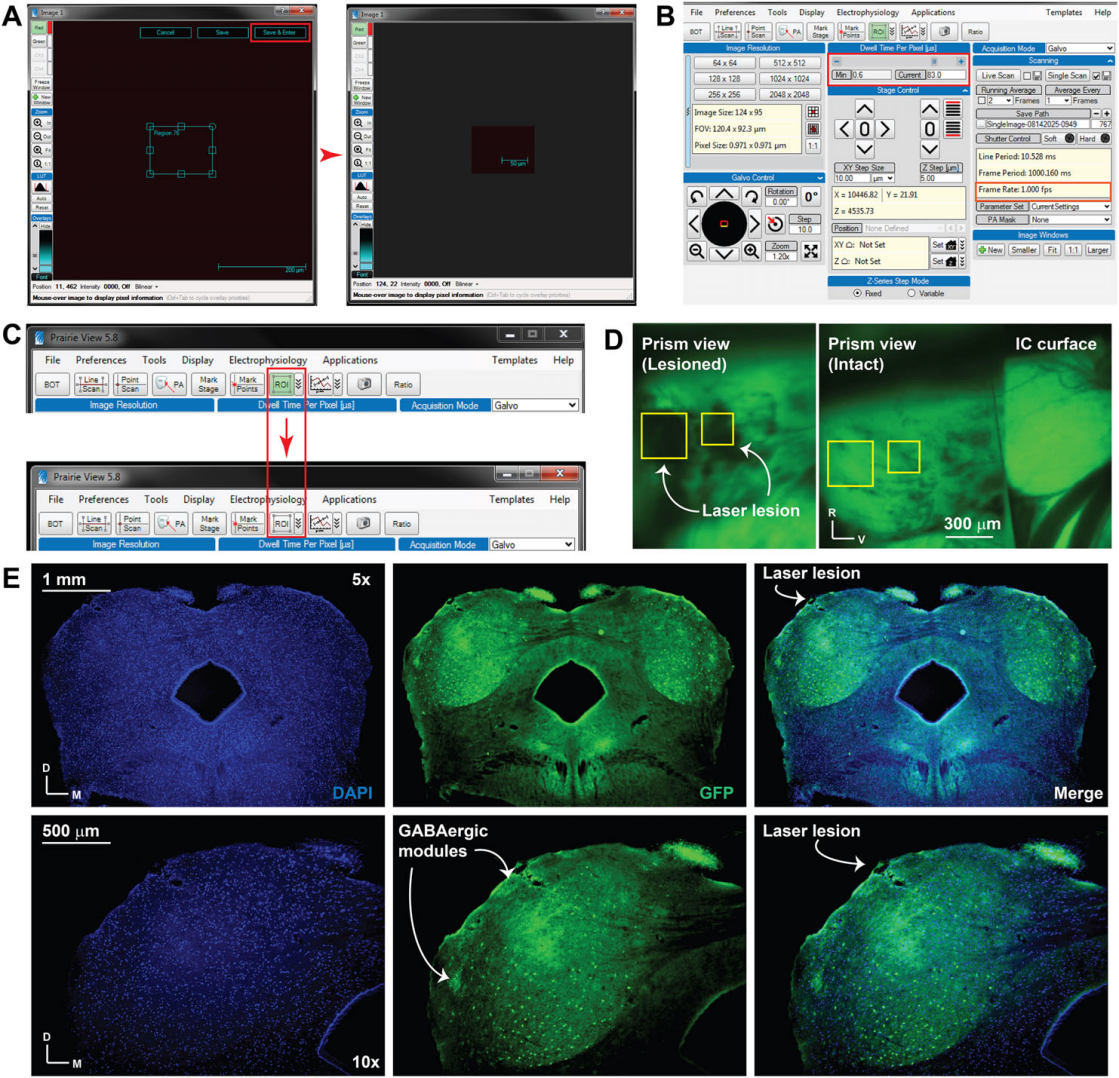
Confirming the microprism location at the LC. (**A**) A screenshot of the Prairie View interface life view window showing the setup to determine the size of the ROI for the laser lesion. The red box indicates where to click to save and enter the icon to approve the selected ROI. (**B**) A screenshot of the Prairie View interface window showing how to control the scan speed by modifying the dwell time per pixel (red box), so that the frame rate is one frame per second (orange box). (**C**) A screenshot of the Prairie View interface window, showing how to deactivate the ROI mode to return to the entire field of view by clicking the active ROI icon to make it inactive (red box and red arrow). (**D**) The prism view images before and after the laser lesion (two spots, yellow boxes). (**E**) Histological examination of the IC coronal sections indicated by DAPI and GFP signals with 5× and 10× objectives, showing the laser lesion at the LC surface.

1 In a galvo acquisition mode with a 920 nm laser and 16× objective, assign and save an ROI for the laser lesion. This ROI should be centered in the field of view and cover 10% of it (Fig. 3A). Once you adjust the ROI size, save and select the ROI so the microscope scans only the selected ROI (Fig. 3A, red box).

2 Use the slider and reduce the dwell time of the laser scan (Fig. 3B‐red box) to scan one frame per second (Fig. 3B orange box).
3 Switch to a high‐power 720 nm laser, use 30% of its power, and begin scanning for 30 s. During scanning, turn on the Chroma barrier et525/70m filter to oversee the laser lesion and stop the process if the laser lesion fills the ROI to avoid over lesioning.
4 Switch back to the 920 nm laser and deactivate ROI selection by clicking its icon to remove the green color (Fig. 3C).
5 Examine the entire cranial window with a 4× objective to detect a small hole in the tissue, as previously done (Fig. 2C), and locate the laser lesion on the prism view. In the experiment shown here, we created two laser lesions (Fig. 3D, yellow boxes).
6 If no lesion is present, increase the time of laser scanning using a 720 nm laser (Add 1 min each time), increase the laser power (add 10% extra each time), or increase the dwell time of laser scanning. You may repeat the procedures until you make a visible lesion.

#### Histological examination of the laser lesion of the LC

7 Prepare for cardiac perfusion using 1× PBS and 4% PFA in 1× PBS.
8 Under deep anesthesia, move the mouse to a dedicated surgical platform for cardiac perfusion, place it supine, and secure it to the platform's base.
9 Make a longitudinal cut along the abdomen, and a transverse cut at the level of the diaphragm. Lift the rib cage so that the heart can be easily exposed. Hold the rib cage in its place by clamping it using the Student Hose Clamp.
10 Make a small cut on the right atrium with the fine scissors. Inject the left ventricle with a 27G needle to deliver 50 ml of 1× PBS, or continue until a clear solution is obtained from the right atrium, to clear the blood from the body.
11 Switch the injection solution to 25 ml of 4% PFA in PBS, or until a sign of complete perfusion is observed. The stiffness of the entire mouse body indicates complete perfusion of the PFA.
12 After removing the head post and the glass components (cover glass and microprism), isolate the brain from the skull and keep it in 4% PFA in PBS for 48 hr at 4°C.
  Because the microprism may be contaminated with PFA, we cannot reuse it from this experiment.
13 Replace the PFA with a sucrose gradient (10%, 20%, and 30% in 1× PBS) as one gradient per day, ensuring that the whole brain is saturated at the end of each day.
14 After a complete brain sink, place the brain into a plastic mold chamber on its anterior axis with super glue.
15 Prepare the egg yolk embedding medium by mixing 1.0 g sucrose with the yolk of one egg. After 10 min, add 600 µl of 25% glutaraldehyde. After 5 s of mixing, quickly pour the mixture to cover the whole brain from all sides.
  The embedding medium will harden within seconds. Therefore, you must act quickly to cover the brain sample with the embedding medium while it is in its liquid form.
16 Place the plastic chamber in a refrigerator for 5 min, then cut away the excess embedding medium around the brain to create a block that includes the brain.
17 Move the brain block to the cryostat holder and attach using O.C.T. compound. Keep the brain block inside the cryostat at −20°C until the whole block is frozen.
18 At the IC level, cut 50‐µm sections and transfer them to a clean slide immediately after cutting to keep the IC tissue intact.
  Refrain from using a floating section technique. The sections may be fragile at the site of the laser lesion, so it is best to place them on a slide immediately. Putting them in PBS could result in greater damage and loss of some section features.
19 Air‐dry the sections away from direct exposure to ambient light, then mount with Vectashield medium containing DAPI. Cover the sections with a coverslip.
20 Examine the GFP signal using a Photometrics (USA) filter with excitation at 488 nm and emission at 515–550 nm, on a single‐photon fluorescent microscope.
  As shown in (Fig. 3E), the microprism implantation caused no damage to the whole structure. However, the laser lesion can be observed in one of the GABAergic modules or in a nearby module.

## Basic Protocol 4 ANALYZING THE TIME TRACES OF NEURONAL RESPONSES AND GENERATING CELL‐RESPONSE MAPS

### Materials

Data, which includes the movies collected after imaging calcium signals following sound playback.
a computer having the following software
  a) Microsoft Windows
  b) Python
  c) ImageJ
  d) Origin Pro
  e) Adobe Photoshop

#### Making the ROIs

**Figure 4:**
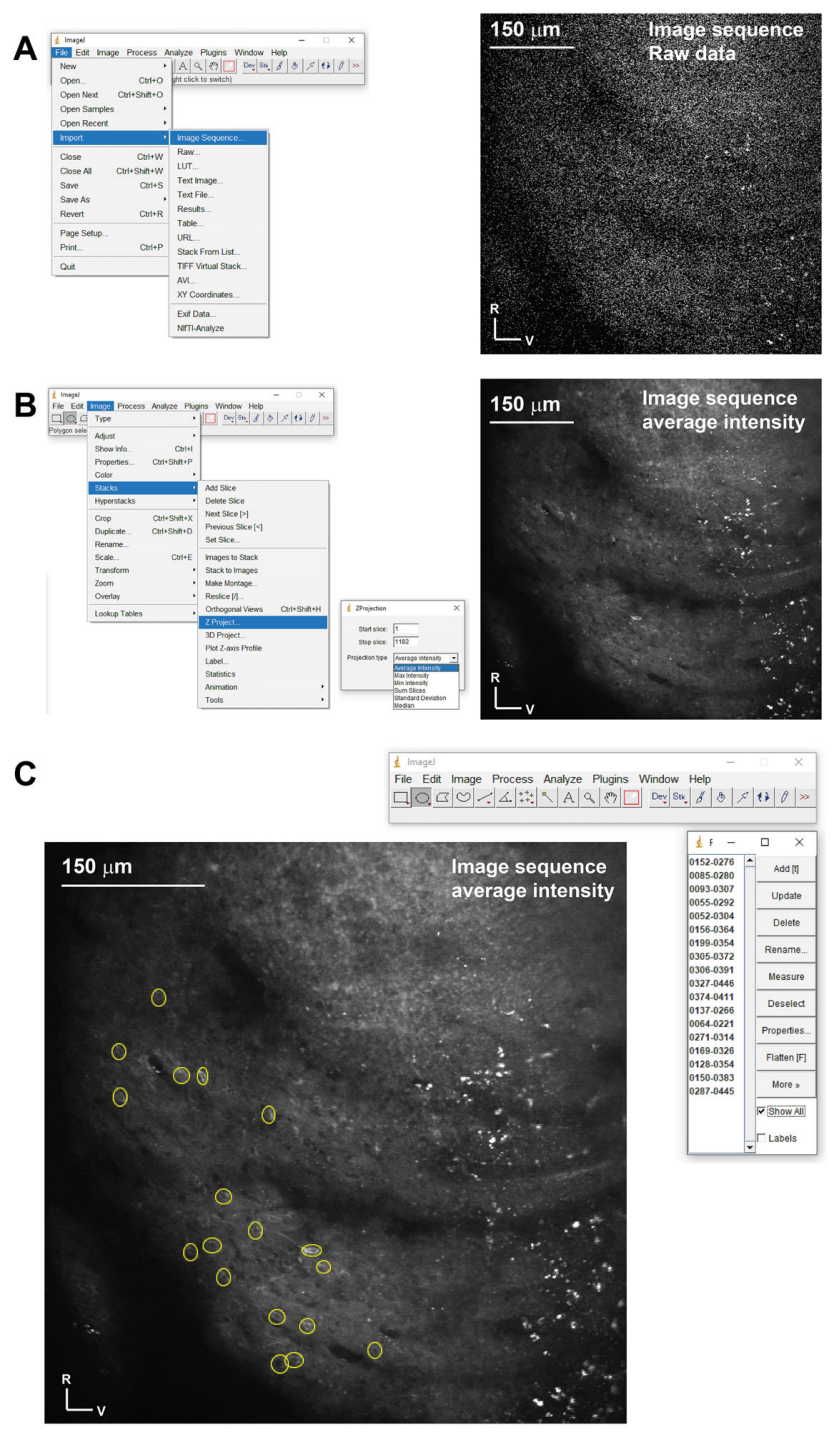
Manually marking the ROIs around cells. (**A**) Left: A screenshot of the ImageJ software showing the steps to open the image sequence (frames) of the movie. Right: an example of the image sequences before averaging. (**B**) Left: A screenshot of the ImageJ software illustrating how to create an average image of the entire movie to enhance cell visualization. Right: an example of the image sequences after averaging. (**C**) A screenshot of the ImageJ software showing examples of the ROIs created by the ROIs manager using an image sequence example and saved as an ROIs zip folder.

1 Import the image sequence of one of the collected movies using ImageJ, following the sequence of commands (File > Import > Image sequence) (Fig. 4A).
  The movies show the calcium signals following sound playback (jRGECO1a signals (red channel)).

2 After ImageJ opens the image sequence as a movie, apply a Z‐project and select a Z‐stack to generate an average intensity image of the entire sequence by following these steps (Image > Stacks > Z Project > (projection type: average intensity)) (Fig. 4B).
  This is a critical step for visualizing neuron cell bodies. Given that the individual frames were collected in a high‐speed resonant Galvo mode, the spatial resolution in each frame is poor.
3 Draw a circle around each neuron and press the letter “t” on the keyboard each time to add the drawn circle as a new entry in the ROI manager (Fig. 4C).
4 Repeat until you collect the ROIs for all cells in the field of view.
5 In the ROI manager, press the “More” icon, then select “Save” from the drop‐down menu to save the ROIs in a zip folder at the preferred location on your computer.

#### Data analysis and the generation of cell‐response maps

6 In one folder named “Data”, all movies should be saved along with the key Excel file that shows the pseudorandom orders of the frequency/amp combinations and the frame number at which each combination starts, as well as the ROI zip folder.
7 Using the Spyder platform, open your customized Python code to run the analysis.
  A free copy of our code is located in the Dryad database. Visit the site of the published database (https://zenodo.org/records/14946966).
8 Run the code to execute the following steps, which will generate the analysis output in an output folder.
  a. A motion stabilization (to correct any faults due to motion artifacts)
  b. Data extraction (to extract the time trace for each cell and subtract the neuropil signals from the cell body signals, as well as calculating the area under the curve, peak response, and the best tuned frequency)
  c. Cell flagging (based on the best‐tuned frequency, the code will assign a color for every cell)
  d. Tonotopic map generation (Based on cell flagging, the code generates a pseudocolor image that is equal to the size of the real image, showing the colored circles at the cell locations based on the coordinates obtained from ROIs)
  e. Cell graphing (draw the time trace of each cell for each frequency/amplitude combination, along with the correlation coefficient between the repeats of these combinations).

#### Understanding the output of the analysis

**Figure 5:**
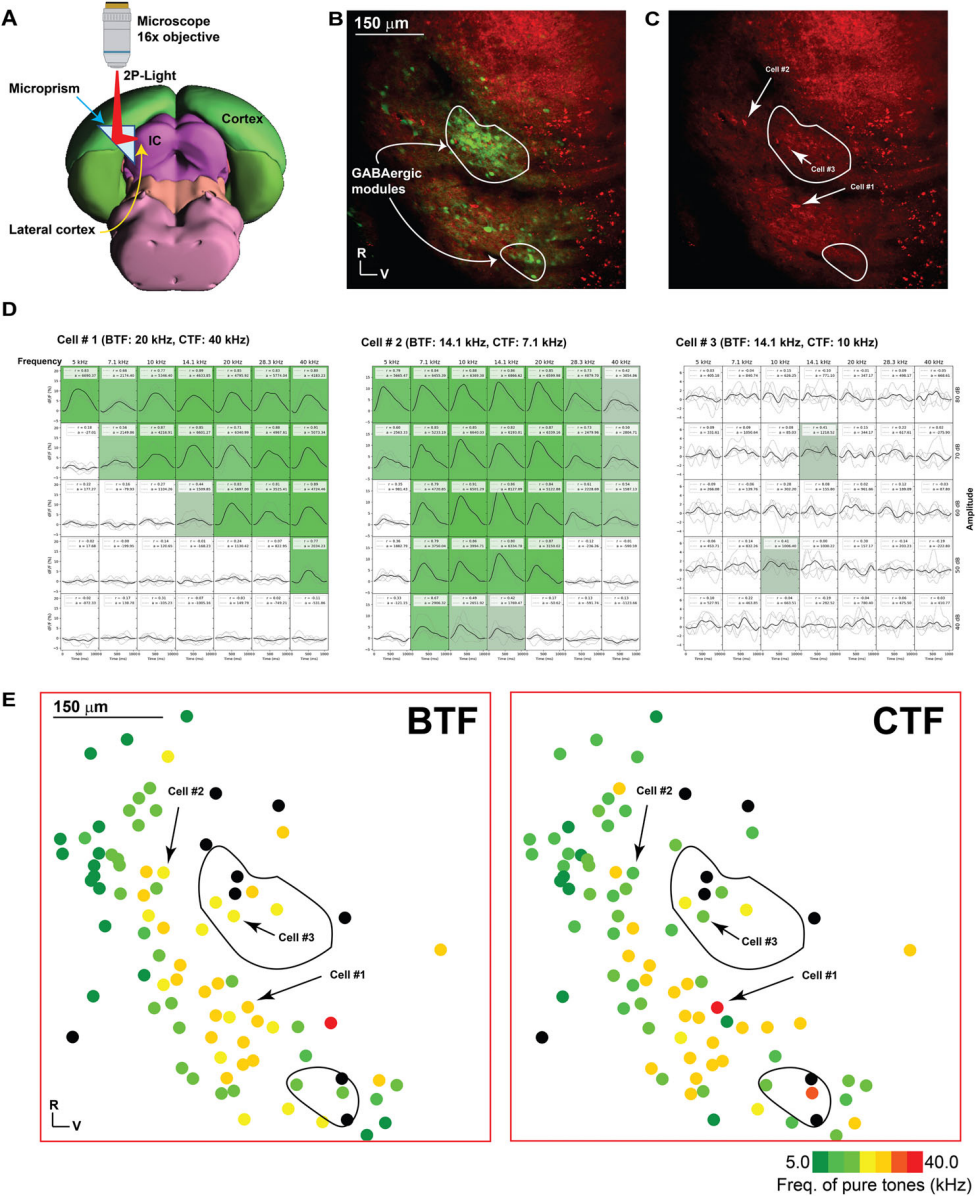
Generating a sound response map for the LC imaged with the microprism. (**A**) A cartoon image showing the mechanism by which the microprism reflects the light to the LC at the sagittal surface. (**B and C**) The LC image obtained using the microprism, showing GFP and jRGECO1a expression. (**D**) The time traces of Ca signals from three different cells in response to different frequency/amplitude combinations of pure tones. (**E**) Two graphs showing the cellular organization map based on BTF or CTF, as indicated in the color legend.

9 After running the code, the output folder should include the following.
  a) A folder having the traces of every cell in one image. Given that there are 35 frequency/amplitude combinations, the image includes 35 spaces in the form of a grid (one space for each combination) (Fig. 5D). In each space, the time traces of the Ca signals for the repeated runs (gray traces), along with the average trace (black trace). The code highlights the space designated to a specific frequency/amplitude combination in green if the correlation coefficient between the repeats (excluding the average) exceeds the assigned threshold, set here to 0.4. Each image displays the numerical values for the best‐tuned frequency (BTF) and the characteristic tuned frequency (CTF) at the top.
**BTF** is the frequency at which the highest average of Ca signals occurs across all sound amplitudes (Barnstedt et al., 2015).
**CTF** is the frequency at which the cell has its detectable Ca signals at the lowest sound amplitude (Wong & Borst, 2019).
  b) Based on the BTF or CTF, the code assigns a color to each cell in the field of view based on its x and y coordinates (Fig. 5E).

## Commentary

### Critical Parameters

Creating a cranial window over the IC is a surgical procedure that requires specialized skills and extensive practice. Given that the IC resides in a highly vascular region, the first goal during surgery is to avoid bleeding. Drilling should be performed under a stream of cold air and cold saline. Skull thickness differs above the IC and its surrounding areas; therefore, the experimenter should avoid puncturing the soft tissues beneath the skull. This can be achieved by drilling at high magnification with the microscope, so that the fine details of the skull can be observed and the bone and soft tissue can be easily distinguished.

Additionally, the experimenter must carefully and gently remove the dura mater, which covers the IC, along with the surrounding main veins. The swift and harsh removal of the dura may result in aggressive detachment and excessive bleeding. Additionally, the dura should be torn only from above the IC, not from above the main vessels located at the midline and sinuses.

The diamond knife is used to facilitate the insertion of a microprism by creating a pocket in the soft tissue. Therefore, the diamond knife should be clean and have a smooth surface. Otherwise, the insertion of the diamond knife can leave scars that result in microbleeding, which can cause minor deformation that may damage the essential characteristics of the LC surface, such as the modules.

The experimenter should minimize manipulation of the IC surface and avoid repeated attempts to insert the diamond knife or microprism. Also, the experimenter should avoid drying the IC surface, which must be kept wet with sterile saline at all times.

The diamond knife and microprism should not be autoclaved, as the high temperature could cause the diamond knife blade to break and deform the microprism structure. These two items should instead be sterilized by exposure to UV light.

During imaging, basal calcium signals should neither be very dim nor very bright. Dim calcium signals indicate loose contact between the orthogonal side of the microprism and the IC tissue. In contrast, bright signals may indicate excessive damage and the presence of dead cells caused by overly aggressive microprism insertion.

### Troubleshooting table

**Table:** 

Problem	Possible cause	Solution
Bleeding	Excessive drilling	Try to stop the bleeding immediately with a saline‐soaked hemostatic sponge. Apply gentle physical pressure to initiate rapid clotting. Pour some cold saline to induce vasoconstriction. With excessive bleeding, you may inject the animal with warm saline (0.1 mL/10 g). Select a sponge that fits only the bleeding site, so the exudate will not spread.
	Aggressive detachment of the bone flap or dura mater	
	Reckless cutting of one of the main veins	
	Aggressive removal of the dura	
No or weak calcium signals obtained	Wrong genotype	Ensure the animal's genotype is accurate
	Blocking the light path	Ensure the laser is turned on and the shutter is open
	Wrong laser or filter	Ensure that the laser and dichroic filter used are compatible with the Ca indicator. In this protocol, a 1040 nm laser is used with the et595/50m filter for jRGECO1a as a Ca indicator.
	Blocked field of view	Check the tissue quality, ensuring it does not contain blood, blood clots, or scars
	Loose contact between the microprism side and the brain tissue.	Ensure the diamond knife is retracted by 0.3 to 0.5 mm so that, when it is pulled off, the microprism remains in place
High‐intensity calcium signals with no sound response	Inflamed area due to excessive manipulation of the surface	Avoid repeated attempts to insert the diamond knife or microprism, as well as excessive cleaning of the surface with a sponge or cotton stick
	Inflamed area due to excessive heat from pouring agarose gel at high temperature	Use agarose gel between (33°C and 37°C)
Good calcium signals, but no sound responses	Imaging of a sound non‐responsive area	Make sure you are at the IC level using the 4× objective
High levels of motion artifact	Mobilized microprism under the round coverslip	Increase the agarose concentration to 2% or less to maintain good visualization and prevent breakage during imaging. 1‐2% agarose is required to retain gel elasticity and transparency.

### Understanding Results

Given that the LC is an inaccessible part of the IC that is covered by brain tissue, the 45° microprism has the power to reflect the excitation and emission light at a 90° angle so that the excitation light can access the LC from the side and the emission light can be received back from the top surface (Fig. 5A – an edited image of the original 3D mouse brain by the Allen Institute), providing a sagittal image of the LC. As indicated by the GFP signals, the image of the LC provided by the microprism revealed the GABAergic modules, which are the most characteristic features of the LC (Fig. 5B). Note that the GABAergic neurons of the IC do not express jRGECO1a, as indicated by the lack of colocalization between GFP and jRGECO1a expression, consistent with previous reports (Dana et al., 2014; Dana et al., 2018) (Fig. 5C). The cells expressing jRGECO1a in the sound‐responsive areas showed transient Ca signals in response to pure tones of different frequency/amplitude combinations (Fig. 5D). Cells located outside the modules have a strong response to sound (Fig. 5D, cell #1 and 2). These two cells exhibit different response profiles to the same frequency/amplitude combinations, consistent with their different BTF and CTF. In contrast, the cells within the modules exhibit a weak response to sound (Fig. 5D, cell #3). However, most modular cells share the same BTF or CTF with those outside the modules (Fig. 5E). The weak sound response of the cells in the modules supports previous studies demonstrating that somatosensory inputs primarily target the modules. In contrast, the area outside the modules, or matrix, is targeted mainly by auditory inputs (Ibrahim et al., 2025; Lesicko et al., 2016). In general, the cellular organization based on BTF or CTF shows that most of the cells are tuned to higher frequencies without showing a tonotopic gradient (Fig. 5E), which supports the previous work showing that the LC is tuned to higher frequencies, which is different from the top surface of the IC, including the DC (Ibrahim et al., 2025).

### Time Consideration

For a skilled experimenter, the surgery may take 90 min. Setting up the animal under the microscope and obtaining the movies for tonotopic organization analysis may take 60 min. Drawing the ROIs, preparing the data for analysis, and the analysis may take 6–10 hr.

### Author Contributions

**Baher Ibrahim**: Conceptualization; data curation; formal analysis; methodology; project administration; software; validation; visualization; writing—original draft. **Daniel Llano**: Conceptualization; funding acquisition; investigation; methodology; project administration; resources; software; supervision; validation; visualization; writing—review and editing.

### Conflict of Interest

The authors declare no conflict of interest.

## Internal Note

All figures and videos are referenced in the main text.

## Data Availability

The data will be available on Dryad, the public database center.
